# Emergency Department Triage Accuracy and Delays in Care for High-Risk Conditions

**DOI:** 10.1001/jamanetworkopen.2025.8498

**Published:** 2025-05-02

**Authors:** Dana R. Sax, E. Margaret Warton, Dustin G. Mark, Mary E. Reed

**Affiliations:** 1 The Permanente Medical Group, Pleasanton, California; 2 The Kaiser Permanente Division of Research, Pleasanton, California

## Abstract

**Importance:**

Emergency department (ED) triage may impact timeliness of care for high-risk conditions.

**Objective:**

To determine the association of ED undertriage with delays in care for patients with subarachnoid hemorrhage (SAH), aortic dissection (AD), and ST-elevation myocardial infarction (STEMI).

**Design, Setting, and Participants:**

This retrospective cohort study included adult ED patients diagnosed with SAH, AD, or STEMI from January 1, 2016, to December 31, 2020, from a multicenter, community-based health care delivery system. Data analysis were completed in March 2023 to October 2024.

**Exposure:**

Undertriage vs correct triage, defined by operational measures of mistriage.

**Main Outcomes and Measures:**

Using a lognormal distribution, the outcomes of interest for patients with SAH and AD were adjusted median time to noncontrast computed tomography (CT) (head CT for patients with SAH, chest CT for patients with AD), antihypertensive medication orders (SAH), and β-blocker orders (AD), and ED length of stay (LOS). For patients with STEMI, outcomes of interest were adjusted median time to electrocardiogram (ECG) and troponin orders.

**Results:**

A total of 5929 patients (median [IQR] age, 63.0 [54.0 to 73.0] years; 3876 [65.4%] male) were identified, including 915 with SAH, 480 with AD, and 4534 with STEMI. There were 1129 Asian patients (19.0%), 553 Black patients (9.3%), 889 Hispanic patients (15.0%), and 2906 non-Hispanic White patients (49.0%). Overall, 2175 patients (36.7%) were undertriaged. For patients with SAH, the lognormal estimate for delay in time to head CT was 0.2 (95% CI, 0.0-0.3), or a delay of 2.4 minutes, and for antihypertensive orders, the lognormal estimate was 4.8 (95% CI, 3.6-5.9), or a delay of 33.3 minutes; the lognormal estimate for ED LOS was 0.1 (95% CI, 0.0-0.1), or 7.7 minutes longer. For patients with AD, the lognormal estimate for delays were 0.2 (95% CI, 0.0-0.4), or 8.9 minutes, for chest CT and 0.5 (95% CI, 0.2-0.7), or 17.6 minutes, for β-blocker orders, and ED LOS was 0.2 (95% CI, 0.1-0.3), or 64 minutes longer. For patients with STEMI, differences in time to ECG and troponin orders were not statistically significant, at less than 1 minute, comparing correctly and undertriaged patients.

**Conclusions and Relevance:**

In this cohort study of patients diagnosed with SAH, AD, or STEMI, ED undertriage was associated with small but significant delays in key diagnostic and therapeutic orders for patients with SAH and AD but not for patients with STEMI.

## Key Points

**Question:**

Is there an association between emergency department (ED) triage accuracy and timeliness of care among patients with subarachnoid hemorrhage (SAH), aortic dissection (AD), or ST elevation myocardial infarction (STEMI)?

**Findings:**

In this cohort study including 5929 patients, undertriage was associated with delayed computed tomography orders by 2.4 minutes for patients with SAH and by 8.9 minutes for patients with AD and delayed medication orders by 33.3 minutes for patients with AD and 17.6 minutes for patients with SAH. Differences in time to electrocardiogram and troponin orders were not statistically significant for undertriaged vs correctly triaged patients with STEMI.

**Meaning:**

These findings suggest undertriaged patients are more likely to experience delays for certain high-risk ED conditions but that consistent and rapid electrocardiogram completion for patients with STEMI likely overrides triage accuracy for these patients.

## Teaser

This cohort study examines the association of undertriage in the emergency department with delays in care for patients with subarachnoid hemorrhage, aortic dissection, and ST-elevation myocardial infarction.

## Introduction

Emergency department (ED) triage, or the sorting of patients based on estimated acuity and resource needs, is necessary to ensure the patients with the most critical needs are treated first. The Emergency Severity Index (ESI), the triage system used in more than 70% of EDs across the US,^1^ uses an algorithm to categorize patients from level I, the most critically ill, to level V, the least critically ill and resource intensive.^2^ Undertriage, or underrecognition of patients with acutely severe illness, may be associated with delays in care because other patients may be prioritized ahead of the undertriaged, contributing to longer wait times.

Certain conditions, including nontraumatic subarachnoid hemorrhage (SAH), aortic dissection (AD), and ST-elevation myocardial infarction (STEMI) rely on timely diagnosis and treatment, and delays in care have been associated with worse patient outcomes.^3,4,5,6,7,8,9,10,11^ For patients with SAH, delays in definitive treatment have been associated with fewer discharges to home (vs rehabilitation in long-term care facilities) and lower survival at 12 months.^8,12^ For patients with AD, studies have estimated a mortality rate of 0.5% to 2% for each hour after symptom onset in proximal thoracic dissections,^9,10,13^ and delays in definitive care are associated with worse outcomes.^11,14^ For patients with STEMI, a longer time to primary percutaneous coronary intervention (known as door-to-balloon time) has been associated with increased risk of short- and long-term mortality, major adverse cardiac events, and reinfarction.^3,4,5,6,7^ Our goal was to describe rates of undertriage among ED patients with a diagnosis of SAH, AD, and STEMI and to assess the associations between undertriage and delays in key ED diagnostic or therapeutic interventions for each condition.

## Methods

This cohort study was approved by the Kaiser Permanente Northern California (KPNC) Institutional Review Board with a waiver for informed consent given the retrospective, data-only study design. The study followed the Strengthening the Reporting of Observational Studies in Epidemiology (STROBE) reporting guideline.

### Design

We applied previously derived operational definitions of mistriage^15,16^ to all patients with an ED diagnosis of SAH, AD, or STEMI to estimate rates of undertriage for each condition. We then used multivariable adjustment to estimate the adjusted times from ED arrival to orders for key diagnostic and therapeutic interventions and total ED length of stay (LOS) for each condition, comparing undertriaged patients with correctly triaged patients.

### Setting and Population

We conducted a retrospective cohort study of patients aged 18 years or older who had an ED visit and received a primary ED diagnosis of SAH, AD, or STEMI between January 1, 2016, and December 31, 2020, across the 21 community EDs in KPNC. We used *International Statistical Classification of Diseases and Related Health Problems, Tenth Revision (ICD-10)* codes (eTable 1 in Supplement 1) to define the study cohort.^17^ KPNC is an integrated health care delivery system that provides comprehensive medical care for more than 4.5 million patients, with approximately 1.2 million ED visits per year. All ED nurses in the study used ESI version 4^16^ during the study period. We excluded encounters with missing ESI or incomplete ED time variables and patients who left the ED against medical advice. Members of KPNC include approximately 33% of the population in areas served and are representative of the demographic and socioeconomic diversity of the surrounding and statewide population.^18,19^

### Variables

We previously created operational definitions of mistriage for each ESI level (eTable 2 in Supplement 1) using electronic health record (EHR) data using a modified Delphi process.^20^ The definitions incorporate occurrence and timing of critical ED interventions and resource use to determine triage accuracy. We applied this algorithm to estimate the frequency of mistriage, which could be undertriage or overtriage, for all study encounters.

We analyzed patient and visit data that we previously found were associated with mistriage,^15^ including demographic information from the EHR and neighborhood socioeconomic status at the census tract level using the most recent American Community Service values, built on 2010 Census data.^21^ We collected race and ethnicity data from the EHR (categorized as Asian, Black, Hispanic, non-Hispanic White, other [including American Indian or Alaska Native, Native Hawaiian or other Pacific Islander, and multiple races or ethnicities], unknown, or missing) to assess disparities. We collected visit time, date, facility, and study year.

We collected history of ED, inpatient, and ICU utilization and information on preexisting illnesses based on history of diagnoses using *ICD-10* codes from the EHR.^17^ For each patient, we obtained an internally derived and validated comorbidity risk score (Comorbidity Point Score, version 2).^22,23^ This risk score is calculated monthly based on the prior 12 months of EHR data and requires KPNC health plan membership during the month in which it is calculated. We used median imputation for continuous variables if there was any missingness, added a flag to indicate observations with imputed values, and included these flags in multivariable models if they were statistically significant. Two authors (D.R.S. and D.G.M.), trained in ESI and blinded to the mistriage algorithm output, manually reviewed 50 encounters, approximately 1% of all study encounters, of which 30 were for STEMI and 10 each were for SAH and AD to assess appropriate labeling of triage accuracy in this cohort.

### Timeliness of Care

We assessed the timing of key diagnostic and therapeutic interventions specific to each condition. We used the time of the order, rather than time of completion, to follow an intention-to-treat protocol. These orders included time to noncontrast computed tomography (CT) head imaging and antihypertensive medication orders for patients with SAH and CT chest imaging with contrast and β-blocker orders for patients with AD. Because not all patients with SAH or AD had orders for these medications, we manually reviewed 25 encounters without orders to ensure electronic capture and to better understand why the treatment may have been withheld. For patients with STEMI, we assessed time to initial electrocardiogram (ECG) and troponin orders. Last, we measured total ED LOS as a measure of time until patient was transferred for definitive care. We did not use ED LOS as an outcome measure for patients with STEMI because catheterization laboratory activation time was included in the mistriage definitions.^15^ Patients admitted to the catheterization laboratory within 1 hour were labeled as undertriaged (if they were not assigned ESI I), thus tying the exposure variable (triage accuracy) with the outcome variable.

### Statistical Analysis

We describe patient and visit characteristics among the full study cohort and separately within each condition. We report frequencies for orders for antihypertensive medications for SAH and β-blockers for AD and ran logistic regression models to assess characteristics associated with orders. Random effects models accounting for clustering by facility and/or patient failed to converge for SAH and AD treatment models, likely due to small numbers of repeated patients.

We used mixed linear models with patient-facility cluster as a random effect to examine the association between undertriage and time to orders and ED LOS, adjusting for patient and visit characteristics. We chose the Cholesky covariance structure to increase model stability and reduce nonconvergence. In cases where the model did not converge due to the number of covariates and a small cohort, we ran simpler regression models that did not account for clustering by patient facility. Because all our time-based measures had a skewed distribution with a long right-tail, we modeled these outcomes assuming a lognormal distribution to avoid biased estimates.

We report the lognormal estimates for each outcome and 95% CIs. In addition, we report least-squares means generated by the LSMEANS method in SAS software version 9.4 (SAS Institute) for the strata in our fully adjusted models. The least-squares means estimates from lognormal models are geometric means, which measure the center of a distribution on an exponential scale and are similar to presenting the adjusted median result at the population level.^24^ All analyses were conducted using 2-sided tests for significance, with *P* < .05 as the threshold for significance. Analyses were performed using SAS software version 9.4.^25^ Data were analyzed from March 2023 to October 2024.

## Results

We analyzed data from 5929 patients (median [IQR] age, 63.0 [54.0 to 73.0] years; 3876 [65.4%] male), including 915 (15.4%) with SAH, 480 (8.1%) with AD, and 4534 (76.5%) with STEMI. The Table presents a description of the study population overall and within each diagnostic group. There were 1129 Asian patients (19.0%), 553 Black patients (9.3%), 889 Hispanic patients (15.0%), 2906 non-Hispanic White patients (49.0%), and 452 patients (7.6%) who identified as other or multiple races. While nearly 3569 patients (78.7%) diagnosed with STEMI presented with chest pain, only 230 patients (47.9%) with AD presented with chest pain, and 419 patients (45.8%) with SAH presented with headache. There were 49 patients (<1%) who were transferred in from another non-KPNC ED.

**Table.** Patient Characteristics Overall and by Presenting Condition and Final Diagnosis

Characteristics	Patients, No. (%)			
	Overall (N = 5929)	By high-risk diagnosis		
		STEMI (n = 4534)	Aortic dissection (n = 480)	Subarachnoid hemorrhage (n = 915)
Age, median (IQR), y	63.0 (54.0 to 73.0)	64 (55.0 to 74.0)	63.0 (53.0 to 75.0)	59.00 (51.0 to 69.0)
Sex				
Male	3876 (65.4)	3215 (70.9)	300 (62.5)	361 (39.5)
Female	2053 (34.6)	1319 (29.1)	180 (37.5)	554 (60.5)
Race and ethnicity				
Asian	1129 (19.0)	830 (18.3)	83 (17.3)	216 (23.6)
Black	553 (9.3)	374 (8.2)	84 (17.5)	95 (10.4)
Hispanic	889 (15.0)	652 (14.4)	51 (10.6)	186 (20.3)
Non-Hispanic White	2906 (49.0)	2327 (51.3)	228 (47.5)	351 (38.4)
Other^a^	452 (7.6)	351 (7.7)	34 (7.1)	67 (7.3)
English is primary language	5354 (90.3)	4119 (90.8)	447 (93.1)	788 (86.1)
Standardized NDI, median (IQR)^b^	−0.24 (−0.72 to 0.40)	−0.24 (−0.73 to 0.39)	−0.17 (−0.69 to 0.56)	−0.27 (−0.72 to 0.36)
Imputed NDI, No. (%)	110 (1.9)	87 (1.9)	10 (2.1)	13 (1.4)
COPS2 risk level^b^^,^^c^				
Low (<20)	3143 (53.0)	2387 (52.6)	214 (44.6)	550 (59.3)
Medium (20 to <65)	2248 (37.9)	1742 (38.4)	205 (42.7)	306 (33.0)
High (≥65)	538 (9.1)	405 (8.9)	61 (12.7)	72 (7.8)
Imputed	961 (16.2)	731 (16.1)	83 (17.3)	150 (16.2)
Past 30 d health care utilization				
Any intensive care	55 (0.9)	36 (0.8)	13 (2.7)	6 (0.7)
Any hospitalization	210 (3.5)	151 (3.3)	26 (5.4)	28 (3.1)
Any ED encounter	543 (9.0)	362 (8.0)	66 (13.8)	108 (11.8)
Past 12 mo health care utilization				
Any intensive care	208 (3.5)	145 (3.2)	36 (7.5)	27 (3.0)
Any hospitalization	626 (10.6)	466 (10.3)	71 (14.8)	89 (9.7)
Any ED encounter	1767 (29.8)	1299 (28.7)	190 (39.6)	278 (30.4)
Study year				
2016	1187 (20.0)	915 (20.2)	84 (17.5)	188 (20.5)
2017	1210 (20.4)	941 (20.8)	99 (20.6)	170 (18.6)
2018	1161 (19.6)	884 (19.5)	96 (20.0)	181 (19.8)
2019	1267 (21.4)	953 (21.0)	110 (22.9)	204 (22.3)
2020	1104 (18.6)	841 (18.5)	91 (19.0)	172 (18.8)
Encounter during office hours (8 am to 4 pm, weekdays)	1852 (31.2)	1408 (31.1)	153 (31.9)	291 (31.8)
Patient arrived by ambulance	2276 (38.4)	1554 (34.3)	153 (31.9)	491 (53.7)
Assigned ESI value				
I	723 (12.2)	581 (12.8)	17 (3.5)	125 (13.7)
II	3967 (66.9)	3219 (71.0)	317 (66.0)	431 (47.1)
III	1236 (20.8)	731 (16.1)	146 (30.4)	359 (39.2)
First or second listed chief complaint				
Chest pain	3815 (64.3)	3569 (78.7)	230 (47.9)	NA
Headache	444 (7.5)	NA	NA	419 (45.8)

Abbreviations: COPS2, Comorbidity Point Score; ED, emergency department; ESI, Emergency Severity Index; NA, not applicable; NDI, Neighborhood Deprivation Index; STEMI, ST elevation myocardial infarction.

^a^ Includes American Indian or Alaska Native, Native Hawaiian or other Pacific Islander, multiple, and unknown or missing.

^b^ Only 2 variables had missingness more than 0.5%: NDI had a missingness rate of 0.8%, whereas COPS2 had a missingness rate of 18.2%. This missingness rate for COPS2 is similar to the rate of patients who were not members of the health care network (19.4%).

^c^ COPS2 is a longitudinal co-morbidity score based on 12 months of patient data.^23^

We found that 1236 patients (20.8%) were assigned a mid- or low-acuity triage assignment (ESI III, IV, or V), with the highest proportion among patients with SAH: 359 patients (39.2%). We estimate that 2175 patients (36.7%) were undertriaged, while 118 patients (2.0%) were classified as overtriaged. In manual EHR review, we agreed with the algorithm’s mistriage categorization in more than 95% of undertriaged and correctly triaged encounters, but in less than 50% of encounters categorized as overtriaged. EHR review revealed that many patients identified as overtriaged received this label because the patient was triaged as ESI level I but was admitted or transferred prior to receiving a critical intervention in the ED that would otherwise have classified them as correctly triaged. For example, patients who had a STEMI diagnosed in the field with a prehospital catheterization laboratory activation were appropriately assigned ESI level I. Some of these patients passed through the ED without any critical interventions. The mistriage algorithm, which uses resource counts and critical interventions to apply mistriage labels, classified these encounters as overtriaged. We excluded these encounters because the label was unreliable and often not representative of true overtriage.

Among patients with AD, 295 (61.5%) had an order for a β-blocker, and among those with SAH, 493 (53.9%) had an order for an antihypertensive medication. We found that orders for a β-blocker were significantly more likely among patients with high (141-180 mm Hg: adjusted odds ratio [AOR], 4.4; 95% CI, 2.5-8.0) or very high (>180 mm Hg: AOR, 9.6; 95% CI, 4.1-22.7) triage systolic blood pressures (compared with patients with blood pressure within reference range) and less likely among patients with low triage heart rates (<60 beats per minute) compared with heart rates with reference range (AOR, 0.3; 95% CI, 0.2-0.6). Antihypertensive medication order was significantly more likely among patients with high (AOR, 5.1; 95% CI, 3.3-8.0) or very high (AOR, 26.4; 95% CI, 14.6-47.6) triage systolic blood pressures (compared with patients with blood pressure within reference range).

We found statistically significant delays in orders and longer ED LOS for undertriaged patients compared with correctly triaged patients with SAH and AD but not STEMI. Among patients with SAH, the lognormal estimates were 0.2 (95% CI, 0.0 to 0.3) for head CT scans, 4.8 (95% CI, 3.6 to 5.9) for antihypertensive medication orders, and 0.1 (95% CI, 0.0 to 0.1) for ED LOS. Among patients with AD, the lognormal estimates were 0.2 (95% CI, 0.0 to 0.4) for chest CT imaging, 0.5 (95% CI, 0.2 to 0.7) for β-blocker orders, and 0.2 (95% CI, 0.1 to 0.3) for ED LOS. Among patients with STEMI, the lognormal estimates were −0.1 (95% CI, −0.2 to −0.1) for time to ECG and −0.0 (95% CI, −0.1 to −0.0) for troponin orders.

**Figure 1.**
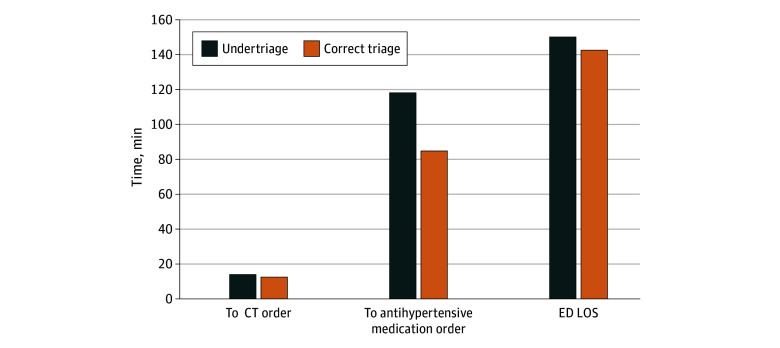
Adjusted Time to Key Diagnostic and Treatment Interventions and Emergency Department (ED) Length of Stay (LOS) Comparing Undertriaged and Correctly Triaged Patients With Subarachnoid Hemorrhage Difference in time to computed tomography (CT) order: *P* = .02; difference in time to antihypertensive order: *P* < .001; difference in ED LOS: *P* = .16. Model adjusted for age; gender; race and ethnicity; primary language; neighborhood deprivation index; active Kaiser Permanente Northern California health plan membership; comorbidity score; ED arrival mode (ambulance vs walk-in); year of visit; time of visit (office hours vs non–office hours); recent ED, inpatient, and intensive care unit utilization; use of anticoagulant; triage vital signs; and chief complaint (headache or no headache). The time to CT model was a mixed-effects model with clustering by facility only. The time to antihypertensive orders model was a mixed-effects model without clustering (models did not converge with any clustering). The ED LOS model was a mixed-effects model with clustering by patient and facility.

**Figure 2.**
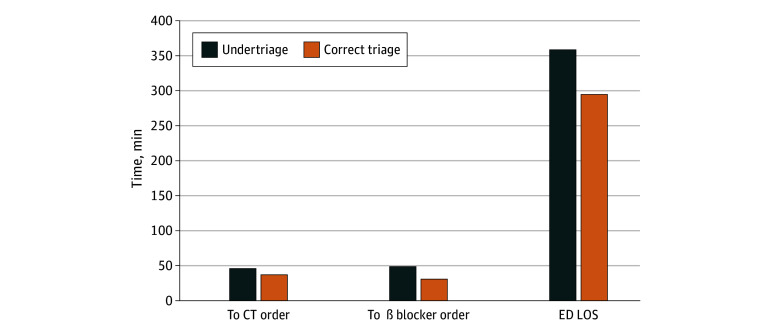
Adjusted Time to Key Diagnostic and Treatment Interventions and Emergency Department (ED) Length of Stay (LOS) Comparing Undertriaged and Correctly Triaged Patients With Aortic Dissection Model adjusted for age; gender; race and ethnicity; primary language; neighborhood deprivation index; active Kaiser Permanente Northern California health plan membership; comorbidity score; emergency department (ED) arrival mode (ambulance vs walk in); year of visit; time of visit (office hours vs non–office hours); recent ED, inpatient, and intensive care unit utilization; use of anticoagulant; triage vital signs; and chief complaint (chest pain or no chest pain). Difference in time to computed tomography (CT) order: *P* = .01; difference in time to β-blocker order: *P* < .001; difference in ED LOS: *P* < .001.

**Figure 3.**
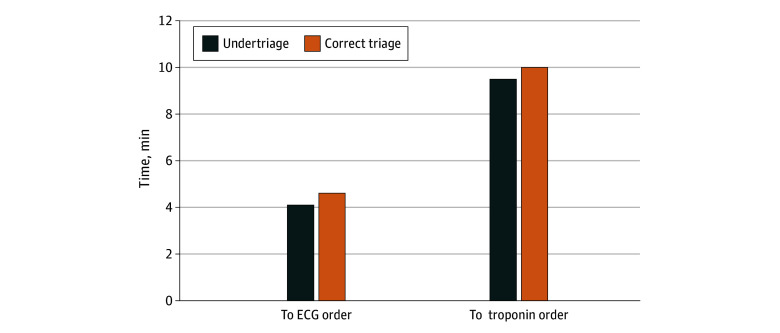
Adjusted Time to Key Diagnostic Interventions Comparing Undertriaged and Correctly Triaged Patients With ST Elevation Myocardial Infarction Difference in time to electrocardiogram (ECG): *P* < .001; difference in time to troponin: *P* = .03. Model adjusted for age; gender; race and ethnicity; primary language; neighborhood deprivation index; active Kaiser Permanente Northern California health plan membership; comorbidity score; ED arrival mode (ambulance vs walk-in); year of visit; time of visit (office hours vs non–office hours); recent ED, inpatient, and intensive care unit utilization; use of anticoagulant; triage vital signs; and chief complaint (chest pain or no chest pain). Both models used mixed effects with clustering by patient and facility.

Figure 1, Figure 2, and Figure 3 show the estimated median times to orders and ED LOS comparing undertriaged patients with correctly triaged patients for each condition. For patients with SAH, undertriaged patients had longer time to CT (15.2 vs 12.8 minutes) and antihypertensive (118.3 vs 85.0 minutes) orders and longer total ED LOS (150.3 vs 142.6 minutes). For patients with AD, undertriaged patients had longer time to CT (46.1 vs 37.2 minutes) and β-blocker (48.3 vs 30.7 minutes) orders and longer total ED LOS (359 minutes vs 295 minutes). For patients with STEMI, differences in time to ECG and troponin orders were less than 1 minute comparing correctly and undertriaged patients.

## Discussion

In this multicenter cohort study of 5929 patients with SAH, AD, and STEMI, ED undertriage occurred in one-third of patients. While prior studies have found that delays in care for patients with SAH, AD, and STEMI are associated with worse patient outcomes,^3,4,5,6,7,8,9,10,11^ as far as we know, this is the first study to look specifically at the association of the triage assessment with ED timeliness of care. In adjusted analyses, we found that undertriage was significantly associated with delays in key diagnostic and treatment orders and time to definitive care for patients with SAH and AD, but not for patients with STEMI.

For patients with SAH, 359 patients (39.2%) received a medium-priority triage assignment (ESI III). After adjustment, undertriaged patients waited an additional 2.4 minutes for CT orders, 33.3 minutes for temporizing medical management (among patients who received an antihypertensive medication), and 7.7 minutes for transfer or admission for definitive care. Our findings are similar to a Canadian study using the Canadian Triage Acuity Score that found a high-acuity ED triage assignment was associated with earlier neuroimaging,^24^ suggesting that undertriage, regardless of the triage algorithm being used, may contribute to delays in care, although the small delay in imaging orders in our study may not be clinically meaningful. We used time to antihypertensive medication order to assess for potential associations between triage accuracy and timely ED care. The evidence to define the balance of potential benefits and risks of blood pressure lowering in the acute phase of SAH is limited,^25,26,27,28,29,30,31,32^ potentially weakening conclusions about the importance of the delay in orders among undertriaged patients.

For patients with AD, we found 146 patients (30.4%) received a medium-priority triage assignment, and, as with patients with SAH, undertriaged patients had significant delays in care. Undertriaged patients waited an additional 8.9 minutes for CT order, 17.6 minutes for temporizing medical management (among patients who received a β-blocker), and 64 minutes for transfer or admission for definitive care. While prior studies have assessed factors influencing timely diagnosis of AD,^33,34^ as far as we know, this is the first to separate out the potential impact of triage assignment.

For patients with STEMI, accurate triage was not associated with more timely care. This may be because the median time to ECG order, the key diagnostic study to identify STEMI, was less than 5 minutes for both correctly and undertriaged patients, well within the American College of Cardiology and the American Heart Association recommendations^35,36^ for ECG completion among patients with possible acute coronary syndrome. All EDs included in this study follow a triage nurse–driven protocol for rapid ECG completion. In addition, some patients are diagnosed before arriving at the hospital with catheterization laboratory activation prior to patient arrival. Although 731 patients (16.1%) with STEMI received a medium-priority triage acuity assignment, rapid ECG completion appeared to be more important than triage assignment in determining downstream timeliness of care.

We previously estimated that mistriage with ESI version 4^16^ occurs in 1 in 3 adult ED encounters^15^ and in 2 in 3 pediatric ED encounters.^37^ As far as we know, this is the first study to evaluate how triage accuracy, after adjusting for patient comorbidities, presenting symptoms, and initial vital signs, may impact how these patients are prioritized. Our findings suggest that accurate triage may contribute to timely care for certain, but not all, high-risk conditions. We hypothesize that mistriage may be similarly associated with delays in care for other high-risk, time-sensitive conditions with protean presentations lacking universal screening studies, such as sepsis, occult trauma, or stroke, although more study is needed.

Accurate triage is particularly challenging for rare diagnoses with frequent atypical presentations. Like other studies,^38,39,40,41^ we found that more than half of patients with SAH and AD had atypical presentations. Prior studies have estimated that atypical presentations contribute to misdiagnosis of AD in 1 in 3 patients,^38^ and up to 1 in 2 patients with SAH.^39,40,41^ Ensuring that every patient with potential SAH or AD is not undertriaged would overwhelm ED resources, although the patients with the highest risk should be prioritized. We previously found that certain clinical characteristics were associated with increased risk of undertriage, including arrival by ambulance, use of certain high-risk medications, greater comorbidity burden, and a recent intensive care unit admission. Identification of these or other high-risk features might improve risk stratification.

Our findings highlight the need to develop, trial, and implement strategies to enhance triage accuracy. Studies suggest that standardized nurse training and regular refresher courses can improve triage accuracy.^42,43,44,45^ In addition, systems that allow for audits, continuous monitoring, and clinician feedback have been shown to improve and maintain triage performance.^42,46,47,48^ Several studies have shown that machine learning–based risk models that incorporate patient EHR data can accurately predict key outcomes at triage, including hospital admission, intensive care unit admission, or death.^49,50,51,52,53,54,55^ These studies have demonstrated that models can achieve high predictive accuracy and improved sensitivity for identifying patients with high risk compared with ESI.^49,50,54^ Access to these risk models at triage, when used to support clinicians in triage, may hold promise in improving accurate identification of the patients with the most critical illness or injury. Recent studies have shown triage models can improve discrimination of patients and efficiency of care,^49,50,56^ although more study of implemented tools is needed to examine their impact on patient outcomes.

### Limitations

This study has some limitations. The main limitation is our retrospective study design and the chance of unmeasured differences between undertriaged and correctly triaged patients or misclassification by triage status. We relied on previously derived and validated comprehensive definitions of triage accuracy.^15^ Prior studies have found high sensitivity and specificity for *ICD-10* codes for STEMI^57^; coding accuracy has been found to be only moderate for patients with AD^58^ and SAH,^59^ and use of *ICD-10* codes may have limited accurate case identification in this study cohort. It is possible that some patients were triaged and then died in the ED before a diagnosis of SAH, AD, or STEMI could be made; these patients would be missed from our analysis. We did not assess how undertriage may have impacted patient outcomes, and this is an important next step in future research. We were limited in our ability to accurately capture door-to-balloon time for patients with STEMI due to significant variation in coding and missingness across sites. We included only time to initial ECG for patients with STEMI. Given ECGs, particularly in STEMI, can be dynamic, mistriage may have had a greater effect among patients with nondiagnostic initial ECGs. We were not able to assess how triage accuracy was associated with ED LOS among patients with STEMI, given that time to cardiac catheterization laboratory activation was used to define undertriage.

There were 49 patients (<1%) who were transferred in from another KPNC facility. Since the diagnosis was already known in these patients, time to treatment orders and ED LOS may have been less, although we do not expect a meaningful effect on overall results, since the number was so small. The last 9 months of the study period occurred during the COVID-19 pandemic, and visit volumes were lower during that time, similar to patterns reported elsewhere due to the COVID-19 pandemic.^60,61^ There were substantial changes in ED volume, acuity, and operational protocols during this period, and we were not able to assess how these changes would have impacted triage accuracy or timeliness of care for patients with STEMI, SAH, or AD. We previously found that the magnitude and factors associated with of mistriage were similar in a large cohort of ED encounters comparing 2016 to 2019 vs 2020, and we hypothesize that findings might be similar in this cohort.^37^ Furthermore, our study findings may not generalize to systems with other protocols for ED triage or treatment of patients with potential STEMI, SAH, or AD.

## Conclusions

In this diverse, multicenter cohort study of nearly 6000 patients with SAH, AD, or STEMI, we found that ED undertriage occurred in more than one-third of patients. For patients with SAH and AD, undertriaged patients were significantly more likely to experience delays in diagnostic and treatment orders and longer total LOS. For patients with STEMI, we did not observe delays in care among undertriaged patients, as rapid ECGs occurred for all patients and likely surpassed the importance of ESI triage assignment in prioritizing care. Our study findings suggest that accurate triage matters for timely ED care, specifically for conditions without a rapid, universal screening test.

## Notes

Supplement 1. **eTable 1.** *ICD-10* Codes **eTable 2.** Definitions of Under- and Over-Triage for Each Emergency Severity Index Level

Supplement 2.
Data Sharing Statement
