# Supplementary material for: Emergency Department Triage Accuracy and Delays in Care for High-Risk Conditions
Source: JAMA Netw Open. 2025 May 2;8(5):e258498. doi: 10.1001/jamanetworkopen.2025.8498 (PMC12048854; doi:10.1001/jamanetworkopen.2025.8498)
Supplement: Supplement 1. — eTable 1. ICD-10 Codes eTable 2. Definitions of Under- and Over-Triage for Each Emergency Severity Index Level [file jamanetwopen-e258498-s001.pdf]

# Supplemental Online Content

Sax DR, Warton EM, Mark DG, Reed ME. Emergency department triage accuracy and delays in care for high-risk conditions. *JAMA Netw Open*. 2025;8(5):e258498.
doi:10.1001/jamanetworkopen.2025.8498

**eTable 1.** *ICD-10* Codes

**eTable 2.** Definitions of Under- and Over-Triage for Each Emergency Severity Index Level

This supplemental material has been provided by the authors to give readers additional information about their work.

© 2025 Sax DR et al. *JAMA Network Open*.

eTable 1: ICD10 codes

| | | | |
|---|---|---|---|
| STEMI | I21.0x | I21.0 | STEMI of anterior wall |
| | | 121.01 | STEMI of left main coronary artery |
| | | I21.02 | STEMI of LAD artery |
| | | I21.09 | STEMI other coronary artery anterior wall |
| | I21.1x | I21.1 | STEMI of inferior wall |
| | | I21.11 | STEMI right coronary artery |
| | | I21.19 | STEMI coronary artery inferior wall |
| | I21.x | I21.2 | STEMI other site |
| | | I21.21 | STEMI circumflex artery |
| | | I21.29 | STEMI involving other sites |
| | I21.3x | I21.3 | STEMI unspecficied site |
| Aortic dissection | | I71.0 | Dissection of aorta |
| | I70.00 | I70.00 | Dissection of unspecified site of aorta |
| | I70.01 | I70.01 | Dissection of thoracic aorta |
| | | I70.010 | Dissection of the ascending aorta |
| | | I70.011 | Dissection of the aortic arch |
| | | I70.012 | Dissection of the descending thoracic aorta |
| | | I70.019 | Dissection of unspecified thoracic aorta |
| | I70.03 | I71.03 | Dissection of thoracoabdominal aorta |
| SAH | I60.0 to 160.9 | I60.0 | Non-traumatic SAH from carotid siphon and bifurcation |
| | | I60.00 | Nontraumatic SAH from unspecified carotid siphon and bifurcation |
| | | I60.01 | |
| | | I60.02 | Nontraumatic SAH from R carotid siphon and bifurcation |
| | | I60.1 | |
| | | I60.10 | Nontraumatic SAH from L carotid siphon and bifurcation |
| | | I60.11 | Non-traumatic SAH from MCA |
| | | I60.12 | Nontraumatic SAH from unspecified MCA |
| | | I60.2 | Nontraumatic SAH from R MCA |

© 2025 Sax DR et al. *JAMA Network Open*.

| | | | |
|---|---|---|---|
| | | I60.3 | Nontraumatic SAH from L MCA |
| | | I60.30 | Nontraumatic SAH from anterior communicating artery |
| | | I60.31 | Nontraumatic SAH from PCA |
| | | I60.32 | Nontraumatic SAH from unspecified PCA |
| | | I60.4 | Nontraumatic SAH from R PCA |
| | | I60.5 | Nontraumatic SAH from L PCA |
| | | I60.50 | Nontraumatic SAH from basilar artery |
| | | I60.51 | Nontraumatic SAH from vertebral artery |
| | | I60.52 | Nontraumatic SAH from unspecified vertebral artery |
| | | I60.6 | Nontraumatic SAH from R vertebral artery |
| | | I60.7 | Nontraumatic SAH from L vertebral artery |
| | | I60.8 | Nontraumatic SAH from other intracranial arteries |
| | | I60.9 | Nontraumatic SAH from unspecified intracranial arteries |
| | | | Other nontraumatic SAH |
| | | | Nontraumatic SAH, unspecified |

Notes: Abbreviations: STEMI, ST elevation myocardial infarction; AD, aortic dissection; SAH, subarachnoid hemorrhage

© 2025 Sax DR et al. *JAMA Network Open*.

eTable 2: Definitions of under- and over-triage for each Emergency Severity Index Level

| Assigned ESI Level | Clinical outcomes – if any occurred, encounter was **under-triaged** |
|---|---|
| V | • Any resource used*<br>• Any hospital admission<br>• Any Level 1, 2, 3, or 4 intervention occurred (see below):<br><br>**Level 1 intervention**: Lifesaving intervention within 1 hour of ED arrival:<br>• Invasive ventilation<br>• Tier I critical medications used: epinephrine, norepinephrine, vasopressin, dopamine, dobutamine, phenylephrine, isoproterenol, atropine, tenecteplase, alteplase<br>• Admit to catheterization suite, operating room, or intensive care unit, or transfer to another hospital<br>• Blood transfusion: fresh frozen plasma, platelets, prothrombin complex concentrate, or ≥ 1 unit packed red blood cells<br>• Death in the ED<br><br>**Level 2 intervention:**<br>• Any of these specific Level 1 interventions beyond first hour: Invasive ventilation, Tier 1 critical medication, admit to catheterization suite, massive transfusion protocol, > 2 units blood, or death in the ED<br>• Any Tier II critical medication used (at any time): nicardipine, sodium nitroprusside, esmolol, milrinone, labetalol drip, nitroglycerin drip, dextrose 50, naloxone, calcium gluconate or calcium chloride, sodium bicarbonate<br>• Parenteral psychotropic medication administered within 120 minutes: haloperidol (≥5mg if patient under 65, ≥2 mg if patient over 65 years), lorazepam (≥2mg), olanzapine (≥10 mg), or ziprasidone (≥10mg)<br>• Non-invasive ventilation<br>• Intraosseous line placed<br><br>**Level 3 intervention:**<br>• Admit to intensive care unit or operating room or transfer to another hospital beyond first hour<br>• Critical procedures: central line, arterial line, paracentesis, thoracentesis, tube thoracostomy, and lumbar puncture<br>• Tier III critical medication used (at any time): Parenteral procainamide, amiodarone, ibutilide, heparin, insulin drip, continuous albuterol<br>• No Level 1 or 2 intervention<br><br>**Level 4 Intervention Critical:**<br>• Tier IV medications used (at any time): Parenteral etomidate, ketamine, Propofol, metoprolol, diltiazem, adenosine, digoxin, hydralazine, or labetalol, or sublingual or transdermal nitroglycerin<br>• 1-2 units packed red blood cell or any other blood product transfusion beyond first hour |

© 2025 Sax DR et al. *JAMA Network Open*.

| | |
|---|---|
| | • No Level 1, 2, or 3 intervention |
| IV | • More than one type resource used<br>• Any Level 1, 2, 3, or 4 intervention occurred (see above)<br>• Admit to hospital (any level of care) |
| III | Any Level 1 or 2 intervention occurred (see above) |
| II | Any Level 1 intervention occurred (see above) |
| Assigned ESI level | Clinical outcomes – if any occurred, encounter considered **over-triaged** |
| I | Less than 2 resources used *and* no Level 1 or 2 intervention occurred (see above) |
| II and III | Less than 2 resources used |
| IV | No resources used |

Notes: Resource use was defined as it is in the Emergency Severity Index, and each different type of resource is counted as a resource, not the individual tests or imaging studies. Resources include: laboratory analysis, EKGs, X-rays, CT, magnetic resonance imaging (MRI), Diagnostic Ultrasound (not point of care), Angiography, IV fluids, IV or IM or nebulized medications. Oral medications, tetanus immunizations, point of care testing, history and physical examination, saline or heplock, prescription refills, simple wound care, crutches, splints, and slings do not count. In our electronic health record, specialty consultation and simple procedures (laceration repair, foley catheter) were not consistently available as discreet fields so these were not counted as resources used.

© 2025 Sax DR et al. *JAMA Network Open*.
