# Supplementary material for: Emergency Department Triage Accuracy and Delays in Care for High-Risk Conditions
Source: JAMA Netw Open. 2025 May 2;8(5):e258498. doi: 10.1001/jamanetworkopen.2025.8498 (PMC12048854; doi:10.1001/jamanetworkopen.2025.8498)
Supplement: Supplement 2. — Data Sharing Statement [file jamanetwopen-e258498-s002.pdf]

Data Sharing Statement

Sax. Emergency Department Triage Accuracy and Delays in Care for High-Risk Conditions. *JAMA Netw Open*. Published May 02, 2025. doi:10.1001/jamanetworkopen.2025.8498

Data
Data available: No
